# Investigation of Age-Related Changes in the Skin Microbiota of Korean Women

**DOI:** 10.3390/microorganisms8101581

**Published:** 2020-10-14

**Authors:** Minseok Kim, Tansol Park, Jung Im Yun, Hye Won Lim, Na Rae Han, Seung Tae Lee

**Affiliations:** 1 Department of Animal Science, College of Agriculture and Life Sciences, Chonnam National University, Gwangju 61186, Korea; mkim2276@jnu.ac.kr; 2 US Dairy Forage Research Center, USDA-ARS, Madison, WI 53706, USA; tansol1719@gmail.com; 3 KustoGen Inc., Chuncheon 24341, Korea; yjros@hanmail.net; 4 Shebah Biotech Inc., Chuncheon 24398, Korea; wendy99cab@gmail.com; 5 Department of Animal Life Science, Kangwon National University, Chuncheon 24341, Korea; hnr327@kangwon.ac.kr; 6 Department of Applied Animal Science, Kangwon National University, Chuncheon 24341, Korea

**Keywords:** 16S rRNA gene, age, forehead, hand, Korean women, skin microbiota

## Abstract

The microbiota of human skin is influenced by host and environmental factors. To determine if chronological age influences the composition of the skin microbiota on the forehead and hands, 73 Korean women were sorted into one of three age groups: (1) 10–29 years (*n* = 24), (2) 30–49 years (*n* = 21), and (3) 50–79 years (*n* = 28). From the 73 women, 146 skin samples (two skin sites per person) were collected. 16S rRNA gene amplicon sequencing was then conducted to analyze the skin microbiota. The overall microbial distribution varied on the forehead but was similar on the hands across the three age groups. In addition, the composition of the skin microbiota differed between the forehead and hands. Commensal microbiota, such as Streptococcus, Staphylococcus, Cutibacterium, and Corynebacterium, which contribute to maintaining skin health via dominant occupation, were affected by increasing age on forehead and hand skin. Alpha diversity indices increased significantly with age on forehead skin. This study indicates that older people may be more susceptible to pathogenic invasions due to an imbalanced skin microbiota resulting from age-related changes. The results of our study may help develop new strategies to rebalance skin microbiota shifted during aging.

## 1. Introduction

Human skin plays an important role in protecting the body against infections by pathogens and harbors a diverse skin microbiota composed of bacteria, archaea, fungi, and viruses, of which bacteria are the most dominant [1,2,3,4]. Balanced colonization of the normal skin microbiota contributes to inhibiting adhesion of pathogens, whereas imbalanced colonization of an abnormal skin microbiota can lead to skin diseases/disorders [4,5,6]. Previous studies have reported microorganisms on the skin that are beneficial [7,8] or detrimental [9,10] to the physiological activity of cells [1,11], and a variety of physiological alterations triggered in the skin can be explained based on this information [12,13,14,15,16]. Accordingly, alterations in microbial communities consisting of skin microbiota have been implicated in the diversification of skin physical or physiological properties and the occurrence of skin-associated diseases, such as acne [15], atopic dermatitis [14], psoriasis [12], and dandruff [13]. Moreover, information on the skin microbiota derived from individuals can play a pivotal role in preparing crucial alternatives for improving personal skin health.

Traditionally, the skin microbiota has been investigated using culture-dependent methods, but most organisms of the skin microbiota cannot be cultured due to the limitations of artificial culture media, resulting in biased results [1]. Since 16S rRNA gene-based next-generation sequencing has been applied to skin microbiota analyses, the taxa of the skin microbiota have been identified with excellent coverage using bioinformatic programs [17,18]. The culture-independent sequencing method has greatly contributed to better understanding of the human skin microbiota and the influence of various factors.

The composition of the skin microbiota is affected by host factors, such as skin site [19], sex [20], immune status [21], and skin disease [4]. The skin microbiota composition is also influenced by environmental factors such as hygiene/cosmetics [22], lifestyle [23], and geography [24]. The skin, a thin layer of soft and flexible tissue forming the integument of the body, is easily exposed to these external factors, resulting in alterations in microbial communities on the skin.

Skin aging starts from birth, and visible signs are observed with advancing age. Skin aging is influenced not only by intrinsic factors such as physiological changes that result in spots, wrinkles, and thin or dry skin, but also by extrinsic factors such as pollution, poor nutrition, and exposure to sun [25]. Age-related changes in the skin microbiota have been observed in Japanese [26] and Chinese women [27]. In addition, the composition of the skin microbiota was found to differ between younger and older Western European women [28]. These three recent studies have revealed that the community structure of skin microbiota is shifted during chronological aging in adult women. The skin microbiota is also affected by different skin locations [2]. Since the forehead and hand are grouped into oily and dry sites respectively, different physiology between these two sites can affect the composition of skin microbiota [1]. In addition, the microbiota of hands is important in the field of hygiene due to its microbial transfer to other body sites, including the forehead site [29].

Since the composition of the skin microbiota is affected by race/ethnicity [30], results obtained from one race/ethnicity cannot be applied to others to improve skin health and well-being. Therefore, race/ethnicity needs to be considered for the development of new skin care products that can efficiently rebalance skin microbiota shifted during chronological aging. Nonetheless, the skin microbiota of Korean people across different age groups and on different skin locations remains unknown. This study investigated the community structure of the skin microbiota on the forehead and hand among Korean people of different ages and evaluated the differences in the skin microbiota between forehead and hand skin using next-generation sequencing.

## 2. Materials and Methods

### 2.1. Experimental Design, Sampling, and Sequencing

This study was approved by the internal review board (KWNUIRB-2019-12-004-001), and all participants in this study submitted written informed consent and declared to be infected with no skin disease and not to be exposed to antibiotics or antifungals for at least 1 month before sampling. A total of 73 Korean women that are urban population in Seoul, the South Korean capital, were sorted into one of three age groups: (1) 10–29 years (*n* = 24), (2) 30–49 years (*n* = 21), and (3) 50–79 years (*n* = 28). From the 73 women, 146 skin samples were collected (two skin sites per person) by swabbing the forehead (a 4 × 2 cm area of the center) and the palm of the hand using a sterile swab kit (KustoGen Inc., Chuncheon, Korea) after removing their makeup. Metagenomic DNA was extracted from the skin samples using the Nucleospin^®^ Tissue XS kit (MACHEREY-NAGEL, Düren, Germany). The V3–V4 region of 16S rRNA genes was sequenced on the Illumina MiSeq platform (Macrogen, Daejeon, Korea). The raw pair-ended amplicon sequence reads were deposited in the NCBI Sequence Read Archive under BioProject PRJNA650212.

### 2.2. Metataxonomic Analysis of Skin Microbiota

The raw 16S amplicon sequences were processed and further analyzed using QIIME2 (version 2019.4) [31]. Preprocessing of the sequence reads was conducted using the DADA2 plugin [32], which performs adapter removal, quality filtering (Q-score cutoff of 25), denoising, merging, and chimeric-sequence removal, simultaneously. The resulting high-quality sequences, called amplicon sequence variants (ASVs), were used for diversity and taxonomic analyses. The Greengenes (v. 13_8) [33] taxonomy classifier, which was pre-trained on the primer set we used to generate phylogenetic amplicons, was clustered at 99% similarity and was used to taxonomically classify ASVs. Additionally, a Venn diagram visualized in R (v. 3.5.0) was used to show the presence and absence of detected taxa at each taxonomic level according to the skin location and three age groups.

The raw ASV count Biological Observation Matrix (BIOM) table was further rarefied at the lowest sequencing counts among the analyzed samples (17,440 counts) before the diversity analysis. Alpha diversity measurements, including richness (observed ASVs and Chao1 estimates), evenness, Faith’s phylogenetic diversity, Shannon’s index, and Simpson’s index, were calculated based on the rarefied ASV BIOM tables. The beta diversity of the skin microbiota according to each variable, including location (forehead vs. hand) and age group (10–29, 30–49, and 50–79 years), was analyzed using principal coordinate analysis (PCoA) based on the unweighted (qualitative) and weighted (quantitative) UniFrac distance matrices.

### 2.3. Functional Predictions Based on Skin Microbiota Community Profiles

To predict the functional composition of the skin microbiota, Phylogenetic Investigation of Communities by Reconstruction of Unobserved States 2 [34] was used to provide MetaCyc pathway profiles predicted from 16S ASVs. Briefly, the ASVs were placed into the reference multiple-sequence alignment followed by placing ASVs into a reference tree. Using the tree file as input, gene family copy numbers of each ASV were predicted with pre-calculated ‘Enzyme Classification (EC) numbers’ and ‘Kyoto Encyclopedia of Genes and Genomes (KEGG) orthologs’ count table. Further normalization of the abundance of individual gene families were computed based on the 16S copy number of each ASV. The MetaCyc pathway abundance was further inferred by regrouping EC numbers, and then the resulting functional profiles were used for the downstream analysis. Overall functional distributions based on the KEGG ortholog profiles were also compared using principal components analysis based on Bray–Curtis dissimilarity matrices.

### 2.4. Statistical Analysis

The linear discriminant analysis (LDA) effect size (LEfSe) [35] was used to identify differentially abundant taxa. MetaCyc pathways were based on the relative abundance of features with an LDA score > 2. For each skin location, the linear and quadratic effects of age on the relative abundances of both the major taxa and functional features were analyzed using orthogonal polynomial contrasts. Permutational multivariate analysis of variance (PERMANOVA) was used to analyze whether the skin location or age group significantly affected the overall distributions of the microbiota or microbial functions, using PAST3 [36] with 9999 random permutations.

## 3. Results

### 3.1. Data Summary

**Figure 1:**
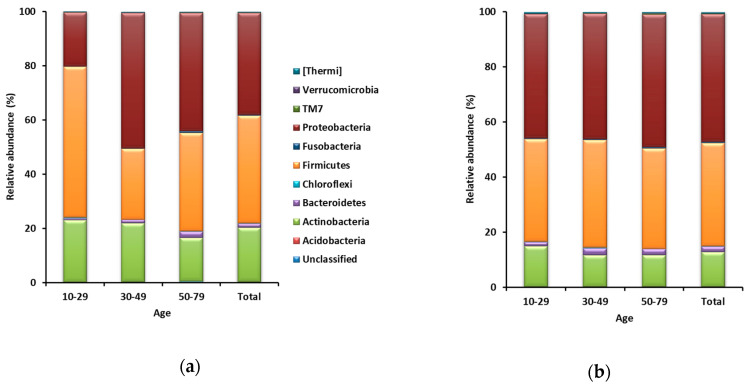
Relative abundances of phyla on both (**a**) forehead skin and (**b**) hand skin.

A total of 6,779,907 sequences were obtained from 146 samples, including 73 forehead and 73 hand skin samples. From each sample, 17,440 sequences (17,440 sequences × 146 samples = 2,546,240 sequences) were randomly sub-sampled for normalization of the number of sequences. Taxa representing >0.5% of the total sequences on average across all 73 forehead or hand samples were regarded as major taxa. The Good’s coverage for all 146 sub-samples was >99%. A total of 31 phyla were identified from the 73 forehead skin samples. Firmicutes was the most dominant and accounted for 39.7% of the total sequences on average across all 73 samples, followed by Proteobacteria (37.6%), Actinobacteria (19.9%), and Bacteroidetes (1.5%) (Figure 1a). Among the 73 hand skin samples, a total of 33 phyla were identified. Proteobacteria was the most dominant and accounted for 46.3% of the total sequences on average across all 73 hand samples, followed by Firmicutes (37.4%), Actinobacteria (12.6%), and Bacteroidetes (2.1%) (Figure 1b).

### 3.2. Differences in Forehead Microbiota among Age Groups

LEfSe showed that Firmicutes was more abundant (*p* < 0.05) in the 10–29-year age group than in the other two age groups (Table 1). In addition, Bacteroidetes and Proteobacteria increased linearly with age (*p* < 0.05) (Table 2). The remaining 27 phyla were represented by <0.5% of the total sequences on average across all 73 forehead samples.

Among the 73 forehead samples, 21 genera were represented by >0.5% of the total sequences on average. Staphylococcus was the most dominant genus and accounted for 27.5% of the total sequences on average across all 73 forehead samples, followed by Pseudomonas (13.4%), Cutibacterium (12.3%), Acinetobacter (4.3%), Bacillus (3.3%), Corynebacterium (3.1%), Streptococcus (2.8%), Xanthomonas (1.9%), Serratia (1.4%), Enterobacter (1.2%), Leuconostoc (1.1%), Stenotrophomonas (1.0%), Enhydrobacter (1.0%), Dietzia (0.7%), Lactobacillus (0.7%), Neisseria (0.7%), Paracoccus (0.7%), Rothia (0.6%), Micrococcus (0.6%), Finegoldia (0.6%), and Haemophilus (0.5%). Among these 21 major genera, Dietzia, Micrococcus, Leuconostoc, Streptococcus, Paracoccus, Acinetobacter, and Enhydrobacter increased linearly with increasing age (*p* < 0.05), while no genera decreased linearly with increasing age (*p* > 0.05) (Table 2).

### 3.3. Differences in the Hand Microbiota among Age Groups

No phylum differed significantly among the three age groups. None of the five major phyla increased or decreased linearly with age. Among all 73 hand samples, 27 genera were represented by >0.5% of the total sequences on average. *Pseudomonas* was the most dominant and accounted for 17.8% of the total sequences on average across all 73 hand samples, followed by Staphylococcus (8.9%), Acinetobacter (7.4%), Leuconostoc (6.6%), Weissella (5.2%), Streptococcus (3.9%), Bacillus (3.5%), Corynebacterium (2.7%), Cutibacterium (2.5%), Lactobacillus (2.1%), Enhydrobacter (1.8%), Rothia (1.2%), Aerococcus (1.1%), Stenotrophomonas (1.1%), Micrococcus (1.0%), Dietzia (0.9%), Erwinia (0.9%), Finegoldia (0.7%), Neisseria (0.7%), Xanthomonas (0.7%), Paracoccus (0.7%), Chryseobacterium (0.6%), Haemophilus (0.6%), Dermacoccus (0.6%), Actinomyces (0.5%), Anaerococcus (0.5%), and Methylobacterium (0.5%). No taxon significantly differed among the three age groups based on the LEfSe analysis. Among these 27 major genera, Leuconostoc increased linearly with increasing age (*p* < 0.05), whereas Corynebacterium, Cutibacterium, Staphylococcus, Weissella, and Xanthomonas decreased linearly with increasing age (*p* < 0.05) (Table 2).

### 3.4. Differences in the Skin Microbiota between the Forehead and Hand Groups

At the phylum level, LEfSe showed that Actinobacteria was significantly more abundant in the forehead group than in the hand group, whereas Proteobacteria was significantly more abundant in the hand group than in the forehead group (Table 3). At the genus level, Cutibacterium and Staphylococcus were significantly more abundant in the forehead group than in the hand group, whereas Acinetobacter, Weissella, Leuconostoc, and Pseudomonas were significantly more abundant in the hand group than in the forehead group (Table 3).

**Figure 2:**
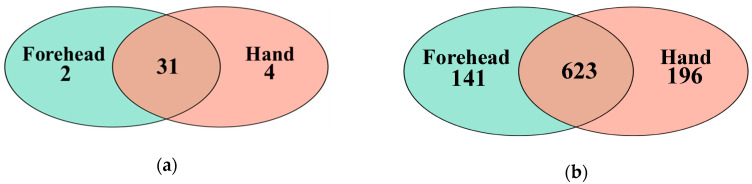
Shared taxa at the phylum and genus levels shown in a Venn diagram. (**a**) A total of 31 phyla were shared between the forehead and hand groups, (**b**) 623 genera were shared between the forehead and hand groups.

The Venn diagram showed that the forehead and hand skin groups shared 31 phyla and had 2 and 4 unique phyla, respectively (Figure 2a). At the genus level, the forehead and hand skin groups shared 623 genera and had 141 and 196 unique genera, respectively (Figure 2b).

### 3.5. Alpha and Beta Diversity Analyses

At least 36,710 sequences on average from all age groups were obtained after quality- and taxonomy-filtration procedures (Table S1). Diversity analysis was conducted by rarefied BIOM tables using the lowest sequencing depth to normalize the number of ASVs per sample. Good’s coverage was >99.2% for all samples. All diversity indices in forehead skin increased linearly with age, whereas no diversity index increased or decreased linearly with age in hand skin (Table 4).

**Figure 3:**
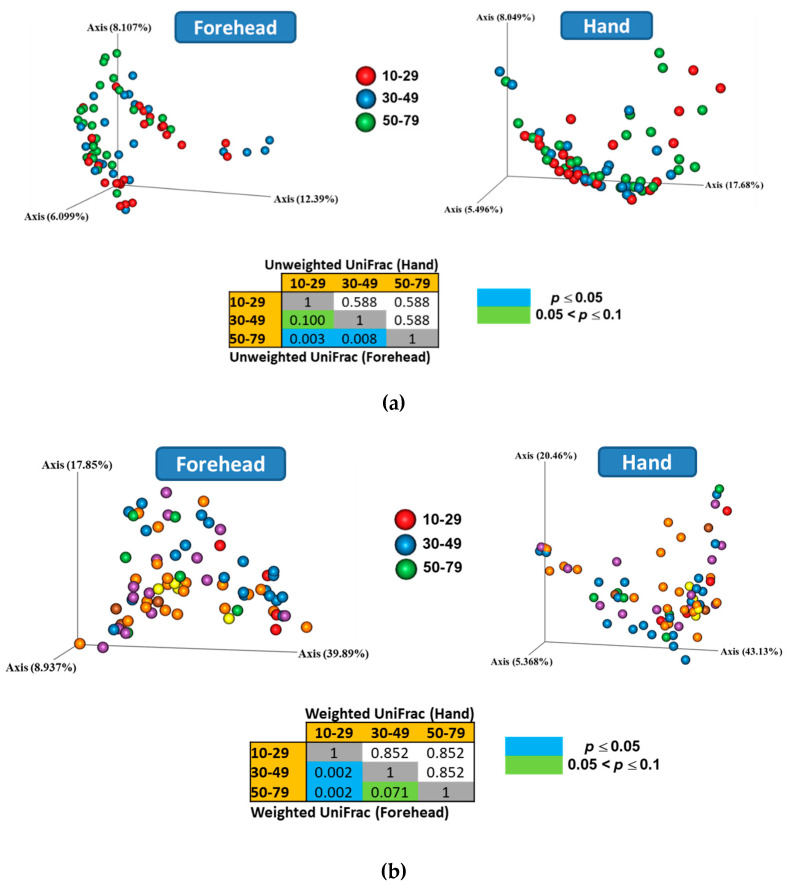
Skin microbiota distribution among the age groups based on the (**a**) unweighted and (**b**) weighted UniFrac distances. Non-parametric Permutational Multivariate Analysis of Variance (PERMANOVA) was used to compare the skin microbiota among age groups.

**Figure 4:**
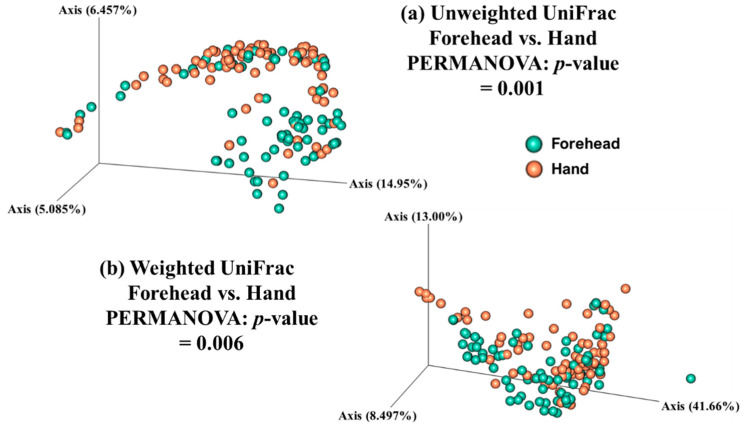
Skin microbiota distribution between the forehead and hand based on the (**a**) unweighted and (**b**) weighted UniFrac distances. Non-parametric Permutational Multivariate Analysis of Variance (PERMANOVA) was used to compare the skin microbiota between the forehead and hand.

Both weighted and unweighted Principal Coordinates Analysis (PCoA) plots showed that the composition of the skin microbiota on the forehead (but not the hand) differed among the age groups (Figure 3). In addition, the PCoA plots showed that the skin microbiota differed between the forehead and hand groups based on both unweighted and weighted UniFrac distance matrices (Figure 4).

### 3.6. Predicted Functional Genetic Profiles

**Figure 5:**
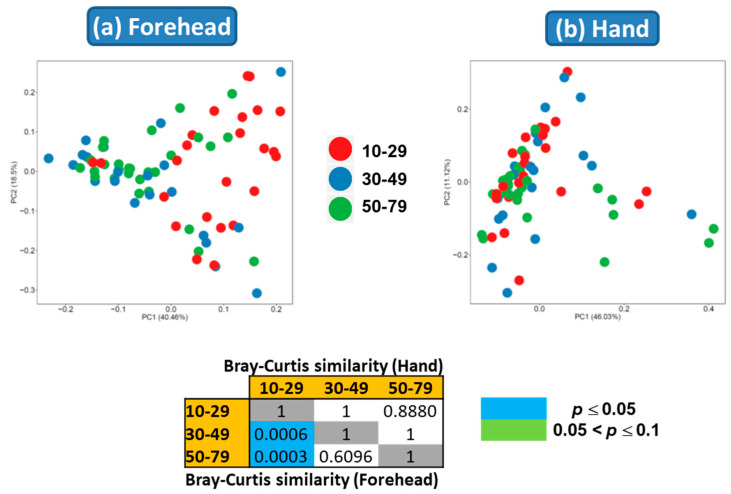
Functional distribution of Kyoto Encyclopedia of Genes and Genomes (KEGG) orthologs on the (**a**) forehead skin and (**b**) hand skin among age groups. Non-parametric Permutational Multivariate Analysis of Variance (PERMANOVA) was used to compare the function distribution of KEGG orthologs among the age groups.

Overall functional genetic profiles were differentially abundant in forehead skin among the different age groups based on PERMANOVA (Figure 5a). In forehead skin, MetaCyc pathways related to gluconeogenesis I (GLUCONEO-PWY) and tetrapyrrole biosynthesis I (PWY-5188) were more predominant in the 10–29-year age group than in the other two age groups (LDA score > 2.5) (Table 5). However, the functional genetic profiles of hand skin did not differ among the age groups (Figure 5b). No MetaCyc pathways in hand skin differed in abundance among the age groups. In both forehead and hand skin, most of the major MetaCys pathways that did not differ in abundance among the age groups were involved in various biosynthesis pathways (Supplementary Tables S2 and S3).

## 4. Discussion

Skin harbors microbiota that help protect the skin from invasion by pathogens [1]. Previous studies have reported that the skin microbiome composition is affected by age [26,27]. However, to date, there has been no report of the impact of age on skin microbiome composition in Korean people. Thus, this is the study to evaluate and compare the skin microbiome of Korean women of different ages and may be useful for advancing studies on skin microbiota comparisons among different races/ethnicities.

The present study demonstrated that Firmicutes, Bacteroidetes, Proteobacteria, and Actinobacteria are major phyla on both forehead and hand skin. Previous studies have shown that these four phyla are commonly found on skin irrespective of race/ethnicity [26,27]. However, the relative abundances of these four phyla may be affected by race/ethnicity. On forehead skin, Actinobacteria and Proteobacteria were the most and second-most dominant phyla respectively, among Japanese people [26], whereas Firmicutes and Proteobacteria were the most and second-most dominant phyla respectively, among Korean people in the present study. The abundance of Firmicutes on skin of Korean people may be influenced by dietary lifestyle associated with fermented food, particularly Kimchi, that is widely consumed by Korean people. Lactobacillus, Weissella, and Leuconostoc placed within Firmicutes are dominant genera that greatly contribute to Kimchi fermentation [37]. In the present study, all these three genera were major taxa across all skin samples, while order Lactobacillales within Firmicutes including uncultured lactic acid bacteria was represented by more than 13% of the total sequences on average in collective data. These taxa seem to contribute to the increase in the abundance of Firmicutes. Proteobacteria on forehead skin was more abundant in older than younger people among other races/ethnicities, including Japanese and Western European people [26,28]. It has been reported that commensal skin microbes can inhibit colonization by pathogens by regulating the expression of various immune factors [38]. Since Proteobacteria includes various pathogenic bacteria, a decline in the ability of older people to resist pathogenic invasions may increase Proteobacteria abundance due to the immune system being weakened by an imbalance of commensal skin microbes. However, Proteobacteria remain poorly understood due to a lack of cultured isolates [39], and further studies are needed to isolate and characterize novel cultured Proteobacteria to elucidate their functions.

The overall microbiome composition on forehead skin differed among age the groups according to PCoA. Dietzia was isolated from the skin of a patient with confluent and reticulated papillomatosis [40]. The increase in Dietzia with age indicates that elderly people are less able to protect their skin from pathogenic invasions due to the weakening immune system. Micrococcus, which is commonly found on human skin, rarely causes problems but can cause skin diseases in immunocompromised people [41]. Although Leuconostoc has not been isolated from human skin, this genus has been found in immunocompromised patients with infections [42]. Streptococcus, which is commonly found in healthy skin microbiota, contributes to maintaining skin health, but a change in its abundance may lead to skin disease [43]. Since some Streptococcus species can cause skin disease in humans [44], a higher abundance of Streptococcus with age may indicate that elderly people are more susceptible to pathogenic invasions. Van Rensburg et al. [41] reported that the community structure of skin microbiomes differed between hosts resistant and those susceptible to infection and found that Paracoccus was more abundant in susceptible hosts. The high abundance of Paracoccus in older people in the present study demonstrates that the ability to resist pathogenic invasions may decrease with age. All of these genera seem to be related to the decreased ability of older people to inhibit colonization by pathogens due to the weakening immune system with age [45]. The microbiome composition on forehead skin appears to be affected by changes in the immune system with age [41]. The increase in colonization by pathogens associated with skin diseases in older people may be inhibited through taking drugs or foods resulting in the enhancement of immune system or applying cosmetics based on cosmetic materials or beneficial microbiota-derived metabolites governing unbeneficial microbiota to the skin.

The hand microbiome composition may be more affected by environment than age. In the present study, similar hand microbiomes among the age groups based on PCoA may result from continuous exposure to similar environments in the same geographic region, rather than skin aging, as described previously [29]. Although a previous study suggested that hand microbiome can be used to predict chronological age [46], skin samples in this study were not collected in a particular region. In the present study, skin samples were collected in a particular region that is Seoul, the South Korean capital. Nonetheless, some genera changed with age. Leuconostoc increased with age on hand skin and was also observed on forehead skin. This also suggests that the ability to resist pathogenic invasions may decrease with age, since the abundance of this genus can increase in immunocompromised people [42]. The opposite was true for Cutibacterium [41], indicating that this genus may be more abundant in younger people, who can maintain better skin health [43]. Smeekens et al. [6] reported that Staphylococcus on hand skin was more abundant among healthy people than immune-compromised people. In the present study, the decrease in Staphylococcus with age may also indicate a decreased ability to inhibit colonization by pathogens. Staphylococcus may be used as an indicator of health status independent of environmental impact. Corynebacterium is commonly found in the healthy skin microbiota, but its altered relative abundance in older people may indicate a decline in the ability to maintain skin health [43]. Little research has been conducted on the roles and functions of Weissella in human skin. Weissella has shown probiotic potential to prevent inflammatory skin disease [47]. The decrease in Weissella on forehead skin with age may help decrease the ability of older people to prevent inflammatory skin diseases. Further studies are needed to isolate and characterize Weissella from human skin to elucidate its functions. Although the overall skin microbiota on the hand did not vary greatly, the abundances of some genera that play an important role in maintaining skin health were altered with increasing age, possibly due to a weakening immune system [45]. These genera may be indicators of healthy hand microbiomes. Further studies, including those using other omics techniques, are required to address this question.

Previous studies have shown that the hand microbiome is unique compared with those at other skin sites [29], consistent with the results of the present study showing that the hand microbiome composition differed significantly from that of the forehead. In addition, previous studies have reported that the hand microbiome is more diverse than those at other skin sites [48,49,50]. Similar results were found in the present study, in which hands tended to have higher diversity than that in foreheads.

We found that the alpha diversity indices of forehead skin were higher in older people than in younger people. Similarly, higher alpha diversity indices from the forehead skin of older people have been reported in both Japanese and Chinese populations [26,30]. These similarities may be due to similar lifestyles and living environments in Asia. However, the alpha diversity indices of hand skin were not different among the age groups, indicating that age-related changes in alpha diversity are related to skin location.

Tetrapyrroles play an important role in living systems as cofactors for various enzymes and proteins, and the tetrapyrrole porphyrin, which is produced by Cutibacterium species, may contribute to maintaining skin health [43,51]. The increase in tetrapyrrole biosynthesis in younger age groups may be associated with a higher abundance of Cutibacterium at younger ages. In the present study, the relative abundance of Cutibacterium was over two-fold greater in the 10–29-year age group than in the 50–79-year age group, although the difference between the two groups was not significant. The increase in MetaCyc pathways related to gluconeogenesis in younger people may indicate that the gluconeogenesis pathway is more important for maintaining the skin microbiota of younger people compared with older people. Further omics studies are needed to elucidate the differences in skin microbiota mechanisms among different age groups.

Although the Illumina paired-end sequencing platform (2 × 300 bp) is commonly used for various microbiome studies due to its cost effectiveness and higher throughput, it generates shorter read lengths compared to other NGS platforms. Meisel et al. [18] indicated that the use of the V4 hypervariable region of the 16S rRNA gene resulted in underestimation of some genera of skin microbiota compared to the V1–V3 hypervariable region. Since the amplicon size of the V1–V3 region is longer than that of the V3–V4 region, the merge rate of the paired-end reads is better in the V3–V4 region than in the V1–V3 region. Therefore, we selected the V3–V4 region to better capture the skin microbiota composition with the increased merge rate in the present study. A previous study with skin bacterial mock community has shown that targeting the V3–V4 region resulted in better accuracy based on the expected abundance of known taxa compared to that of the V1–V3 region [17]. Whon et al. [52] reported that the V3–V4 region has a better correlation coefficient with shotgun metagenomic classification than the V1–V3 region (*r* = 0.98 vs. 0.75) regarding major bacterial abundance between the sequence datasets extracted from human feces samples. Therefore, the use of the V3–V4 region seems to be a reasonable approach to investigate the composition of skin microbiota. In addition, Benson et al. [53] indicated that a minimum of 30 sequence reads can be used as a threshold for quantitative repeatability. Since the present study analyzed major taxa representing >0.5% of 17,440 sequences (>87 sequence reads) in each sample, the results in the present study are thought to be reproducible.

The results of the present study demonstrate that the overall microbiome composition was altered on forehead skin with increasing age, while some genera changed during aging on hand skin. The community structure of skin microbiomes was different between forehead and hand sites. Balanced commensal skin microbiota that maintain skin health may be altered by age due to a weakening immune system. The present data elucidate the skin microbiota associated with chronological aging and may provide potential opportunities to develop strategies to prevent skin disorders resulting from chronological aging in Korean women.

## Figures and Tables

**Table 1:** Differentially abundant taxa among age groups.

Location	Feature	10–29 Years	30–49 Years	50–79 Years	SEM ^1^	Class	LDA ^2^ Score	*p*-Value
Forehead	Firmicutes	55.69	26.10	36.23	3.116	10-20s	5.18	0.0021

^1^ Standard error of the mean. ^2^ Linear discriminant analysis.

**Table 2:** Linearly affected major classified taxa by age grouping.

Taxa ^1^	Location	10–29 Years	30–49 Years	50–79 Years	SEM ^2^	Linear	Quadratic
Proteobacteria	Forehead	19.870	50.754	43.565	0.057	<0.0001	0.0055
Bacteroidetes	Forehead	0.679	1.449	2.353	0.096	0.0009	0.3832
Dietzia	Forehead	0.297	0.629	1.136	0.210	0.0033	0.2949
Micrococcus	Forehead	0.153	0.554	1.019	0.196	0.0003	0.7649
Leuconostoc	Forehead	0.023	0.379	2.663	0.980	0.0147	0.6437
Streptococcus	Forehead	1.453	1.719	4.816	0.495	0.0040	0.2980
Paracoccus	Forehead	0.176	0.356	1.336	0.314	0.0005	0.5069
Acinetobacter	Forehead	2.140	5.472	5.267	0.824	0.0371	0.7581
Enhydrobater	Forehead	0.401	1.284	1.221	0.184	0.0331	0.4686
Corynebacterium	Hand	4.010	2.829	1.413	0.345	0.0164	0.8079
Cutibacterium	Hand	3.883	2.759	1.007	0.529	0.0026	0.7473
Staphylococcus	Hand	15.365	7.837	4.255	1.336	0.0091	0.4853
Lactobacillus	Hand	1.517	4.328	0.851	0.700	0.0402	0.9095
Weissella	Hand	6.107	4.780	4.706	2.224	0.0208	0.2133
Xanthomonas	Hand	1.493	0.796	0.021	0.301	0.0021	0.8681

^1^ Taxa representing >0.5% of the total sequences on the average across all 73 forehead or hand samples. ^2^ Standard error of the mean.

**Table 3:** Differentially abundant taxa in different skin locations.

Taxa	Relative Abundance		SEM ^1^	Class	LDA ^2^ Score	*p*-Value
	Forehead	Hand				
Proteobacteria	37.55	46.29	2.163	Hand	4.60	0.0337
Actinobacteria	19.89	12.59	1.167	Forehead	4.54	0.0269
Acinetobacter	4.28	7.42	0.901	Hand	4.23	0.0108
Staphylococcus	27.54	8.98	1.857	Forehead	4.95	<0.0001
Weissella	0.09	5.19	1.129	Hand	4.40	0.0006
Leuconostoc	1.14	6.53	1.130	Hand	4.47	0.0464
Cutibacterium	12.32	2.47	1.037	Forehead	4.67	<0.0001
Pseudomonas	13.41	17.81	1.404	Hand	4.35	0.0483

^1^ Standard error of the mean. ^2^ Linear discriminant analysis.

**Table 4:** Alpha-diversity measurements by age groups.

Forehand						
Age Group	Observed ASVs	Chao1	Evenness	Faith’s PD ^1^	Shannon’s Index	Simpson’s Index
10–29 years	122 ^b^	128 ^b^	0.49 ^b^	11.57 ^b^	3.36 ^b^	0.74 ^b^
30–49 years	155 ^b^	157 ^b^	0.58 ^ab^	14.80 ^ab^	4.00 ^ab^	0.81 ^ab^
50–79 years	247 ^a^	253 ^a^	0.61 ^a^	19.8 ^a^	4.78 ^a^	0.86 ^a^
*p*-value	<0.0001	<0.0001	0.0127	0.0017	0.0012	0.0254
Linear	<0.0001	<0.0001	0.0043	0.0004	0.0003	0.0075
Quadratic	0.3877	0.3351	0.3932	0.8394	0.9865	0.6292
SEM	13.33	13.64	0.02	1.01	0.17	0.02
**Hand**						
**Age Group**	**Observed ASVs**	**Chao1**	**Evenness**	**Faith’s PD ^1^**	**Shannon’s Index**	**Simpson’s Index**
10–29 years	209	217	0.61	23.21	4.64	0.83
30–49 years	195	201	0.62	20.73	4.53	0.82
50–79 years	220	225	0.61	24.17	4.67	0.84
*p*-value	0.8150	0.8376	0.9752	0.5202	0.9655	0.9777
Linear	0.7405	0.8198	0.9722	0.6883	0.9460	0.9565
Quadratic	0.5932	0.5889	0.8264	0.2917	0.8003	0.8396
SEM	15.68	16.36	0.02	1.23	0.21	0.02

^1^ Faith’s phylogenetic diversity. No significantly differed alpha-diversity measurements were found by different skin types (*p* > 0.1) (data not shown). Age groups having differing superscripts are different (*p* < 0.05).

**Table 5:** Differentially abundant major functions within MetaCyc pathways by Age groups.

Feature	10–29 Years	30–49 Years	50–79 Years	SEM ^1^	Class	LDA ^2^ Score	*p*-Value
Gluconeogenesis I	0.60	0.57	0.53	0.009	10–29 years	2.62	0.0001
Tetrapyrrole biosynthesis I (from glutamate)	0.60	0.54	0.50	0.011	10–29 years	2.78	0.0001

^1^ Standard error of the mean. ^2^ Linear discriminant analysis.

## Supplementary Materials

The following are available online at https://www.mdpi.com/2076-2607/8/10/1581/s1, Table S1: DADA2 denoising statistics of 16S rRNA gene sequences. Table S2: Major MetaCyc pathways (0.5% at least one of age groups) by age groups in forehead skin. Table S3: Major MetaCyc pathways (0.5% at least one of age groups) by age groups in hand skin.
