# Supplementary material for: Investigation of Age-Related Changes in the Skin Microbiota of Korean Women
Source: Microorganisms. 2020 Oct 14;8(10):1581. doi: 10.3390/microorganisms8101581 (PMC7602415; doi:10.3390/microorganisms8101581)
Supplement: Supplementary file 1 [file microorganisms-08-01581-s001.pdf]

Table 1. DADA2 denoising statistics of 16S rRNA gene sequences.

Sequence read type	No. of sequence reads[1] 10-29 years (*n*=24)[3]	30-49 years (*n*=21)[4]	50-79 years (*n*=28)[5]	SEM[6]
**Forehead (*n*=73)**				
No. of input paired reads	81,526	59,402	63,603	4,886
Quality filtered reads	68,362	47,898	51,089	4,391
Denoised reads	68,072	47,673	50,627	4,382
Merged reads	64,555	45,495	48,123	4,136
Chimera-filtered sequences	53,989	37,132	43,269	3,914
Taxa-filtered sequences[2]	53,851	36,710	42,794	3,904
**Hand (*n*=73)**				
**Sequence read type**	**No. of sequence reads[1] 10-29 years (*n*=24)[3]**	**30-49 years (*n*=21)[4]**	**50-79 years (*n*=28)[5]**	**SEM[6]**
No. of input paired reads	79,506	58,005	60,967	4,886
Quality filtered reads	64,658	45,419	48,449	4,391
Denoised reads	64,312	45,026	47,995	4,382
Merged reads	61,291	42,813	45,663	4,136
Chimera-filtered sequences	57,765	39,597	42,988	3,914
Taxa-filtered sequences[2]	56,738	37,354	41,681	3,904

[1] Values represent means. [2] ASVs identified as 'mitochondria', 'chloroplast', and 'Unassigned' were filtered out. [3] 10s (*n*=4) and 20s (*n*=20). [4] 30s (*n*=6) and 40s (*n*=15). [5] 50s (*n*=21), 60s (*n*=4) and 70s (*n*=3). [6] Standard error of the mean. A total of 146 skin samples were analyzed (73 people × 2 skin sites = 146 samples).

Table 2. Major MetaCyc pathways (>0.5% at least one of the age-groups) by age groups in forehead skin.

Feature	Relative abundance (%)			SEM	Description
	10-29 years	30-49 years	50-79 years		
1CMET2-PWY	0.482	0.520	0.530	0.008	N10-formyl-tetrahydrofolate biosynthesis
ANAEROFRUCAT-PWY	0.649	0.542	0.524	0.015	homolactic fermentation
ANAGLYCOLYSIS-PWY	0.672	0.565	0.555	0.016	glycolysis III (from glucose)
ARGSYN-PWY	0.566	0.513	0.506	0.009	L-arginine biosynthesis I (via L-ornithine)
ARGSYNBSUB-PWY	0.630	0.558	0.550	0.011	L-arginine biosynthesis II (acetyl cycle)
ARO-PWY	0.601	0.574	0.589	0.005	chorismate biosynthesis I
BRANCHED-CHAIN-AA-SYN-PWY	0.703	0.641	0.661	0.007	superpathway of branched amino acid biosynthesis
CALVIN-PWY	0.576	0.531	0.561	0.006	Calvin-Benson-Bassham cycle
COA-PWY	0.559	0.514	0.530	0.007	coenzyme A biosynthesis I
COMPLETE-ARO-PWY	0.636	0.603	0.620	0.006	superpathway of aromatic amino acid biosynthesis
DAPLYSINESYN-PWY	0.536	0.560	0.551	0.011	L-lysine biosynthesis I
DENOVOPURINE2-PWY	0.599	0.501	0.517	0.013	superpathway of purine nucleotides de novo biosynthesis II
FAO-PWY	0.491	0.561	0.524	0.018	fatty acid β-oxidation I
FASYN-ELONG-PWY	0.587	0.625	0.645	0.015	fatty acid elongation -- saturated
FOLSYN-PWY	0.585	0.567	0.565	0.007	superpathway of tetrahydrofolate biosynthesis and salvage
GLUCONEO-PWY	0.599	0.567	0.529	0.009	gluconeogenesis I
GLYCOLYSIS	0.640	0.554	0.554	0.012	glycolysis I (from glucose 6-phosphate)
HEMESYN2-PWY	0.580	0.531	0.535	0.007	heme biosynthesis II (anaerobic)
ILEUSYN-PWY	0.727	0.668	0.708	0.010	L-isoleucine biosynthesis I (from threonine)
NONOXIPENT-PWY	0.641	0.615	0.620	0.008	pentose phosphate pathway (non-oxidative branch)
PEPTIDOGLYCANSYN-PWY	0.605	0.536	0.553	0.008	peptidoglycan biosynthesis I (meso-diaminopimelate containing)
PHOSLIPSYN-PWY	0.708	0.671	0.697	0.008	superpathway of phospholipid biosynthesis I (bacteria)
PWY-2942	0.680	0.615	0.619	0.008	L-lysine biosynthesis III
PWY-3001	0.647	0.592	0.607	0.006	superpathway of L-isoleucine biosynthesis I
PWY-3781	1.366	1.378	1.278	0.047	aerobic respiration I (cytochrome c)
PWY-5097	0.524	0.552	0.530	0.010	L-lysine biosynthesis VI
PWY-5101	0.763	0.718	0.754	0.007	L-isoleucine biosynthesis II

PWY-5103	0.668	0.612	0.630	0.007	L-isoleucine biosynthesis III
PWY-5188	0.602	0.540	0.498	0.011	tetrapyrrole biosynthesis I (from glutamate)
PWY-5189	0.573	0.512	0.476	0.010	tetrapyrrole biosynthesis II (from glycine)
PWY-5484	0.660	0.552	0.543	0.015	glycolysis II (from fructose 6-phosphate)
PWY-5659	0.480	0.537	0.500	0.011	GDP-mannose biosynthesis
PWY-5667	0.713	0.692	0.739	0.009	CDP-diacylglycerol biosynthesis I
PWY-5686	0.628	0.589	0.604	0.007	UMP biosynthesis
PWY-5695	0.519	0.602	0.594	0.013	urate biosynthesis/inosine 5'-phosphate degradation
PWY-5973	0.589	0.602	0.654	0.012	cis-vaccenate biosynthesis
PWY-6121	0.652	0.593	0.616	0.008	5-aminoimidazole ribonucleotide biosynthesis I
PWY-6122	0.649	0.571	0.600	0.010	5-aminoimidazole ribonucleotide biosynthesis II
PWY-6123	0.560	0.515	0.532	0.007	inosine-5'-phosphate biosynthesis I
PWY-6125	0.647	0.600	0.594	0.008	superpathway of guanosine nucleotides de novo biosynthesis II
PWY-6126	0.661	0.611	0.613	0.008	superpathway of adenosine nucleotides de novo biosynthesis II
PWY-6147	0.649	0.605	0.588	0.010	6-hydroxymethyl-dihydropterin diphosphate biosynthesis I
PWY-6163	0.590	0.554	0.571	0.005	chorismate biosynthesis from 3-dehydroquinate
PWY-6277	0.649	0.571	0.600	0.010	superpathway of 5-aminoimidazole ribonucleotide biosynthesis
PWY-6385	0.536	0.513	0.537	0.009	peptidoglycan biosynthesis III (mycobacteria)
PWY-6386	0.616	0.530	0.556	0.009	UDP-N-acetylmuramoyl-pentapeptide biosynthesis II (lysine-containing)
PWY-6387	0.614	0.541	0.559	0.009	UDP-N-acetylmuramoyl-pentapeptide biosynthesis I (meso-diaminopimelate containing)
PWY-6612	0.574	0.554	0.547	0.008	superpathway of tetrahydrofolate biosynthesis
PWY-6969	0.579	0.557	0.534	0.016	TCA cycle V (2-oxoglutarate:ferredoxin oxidoreductase)
PWY-7111	0.926	0.891	0.928	0.015	pyruvate fermentation to isobutanol (engineered)
PWY-7184	0.600	0.571	0.562	0.012	pyrimidine deoxyribonucleotides de novo biosynthesis I
PWY-7197	0.548	0.500	0.498	0.008	pyrimidine deoxyribonucleotide phosphorylation
PWY-7208	0.730	0.650	0.662	0.010	superpathway of pyrimidine nucleobases salvage
PWY-7219	0.653	0.612	0.624	0.007	adenosine ribonucleotides de novo biosynthesis
PWY-7220	0.674	0.615	0.604	0.010	adenosine deoxyribonucleotides de novo biosynthesis II
PWY-7221	0.637	0.604	0.609	0.008	guanosine ribonucleotides de novo biosynthesis
PWY-7222	0.674	0.615	0.604	0.010	guanosine deoxyribonucleotides de novo biosynthesis II

PWY-7228	0.669	0.624	0.617	0.008	superpathway of guanosine nucleotides de novo biosynthesis I
PWY-7229	0.686	0.637	0.643	0.008	superpathway of adenosine nucleotides de novo biosynthesis I
PWY-7234	0.556	0.511	0.519	0.007	inosine-5'-phosphate biosynthesis III
PWY-7254	0.661	0.589	0.552	0.014	TCA cycle VII (acetate-producers)
PWY-7400	0.564	0.511	0.504	0.009	L-arginine biosynthesis IV (archaebacteria)
PWY-7539	0.653	0.605	0.594	0.010	6-hydroxymethyl-dihydropterin diphosphate biosynthesis III (Chlamydia)
PWY-7663	0.595	0.672	0.699	0.018	gondoate biosynthesis (anaerobic)
PWY-841	0.640	0.590	0.598	0.008	superpathway of purine nucleotides de novo biosynthesis I
PWY0-1319	0.713	0.692	0.739	0.009	CDP-diacylglycerol biosynthesis II
PWY0-1586	0.475	0.532	0.507	0.023	peptidoglycan maturation (meso-diaminopimelate containing)
PWY0-162	0.637	0.590	0.595	0.008	superpathway of pyrimidine ribonucleotides de novo biosynthesis
PWY0-166	0.559	0.530	0.529	0.011	superpathway of pyrimidine deoxyribonucleotides de novo biosynthesis (E. coli)
PWY4FS-7	0.670	0.624	0.659	0.008	phosphatidylglycerol biosynthesis I (plastidic)
PWY4FS-8	0.670	0.624	0.659	0.008	phosphatidylglycerol biosynthesis II (non-plastidic)
REDCITCYC	0.656	0.579	0.529	0.015	TCA cycle VIII (helicobacter)
RIBOSYN2-PWY	0.572	0.527	0.524	0.008	flavin biosynthesis I (bacteria and plants)
SER-GLYSYN-PWY	0.659	0.663	0.648	0.009	superpathway of L-serine and glycine biosynthesis I
TCA	0.746	0.689	0.629	0.013	TCA cycle I (prokaryotic)
THRESYN-PWY	0.616	0.563	0.576	0.006	superpathway of L-threonine biosynthesis
TRNA-CHARGING-PWY	0.532	0.528	0.524	0.009	tRNA charging
TRPSYN-PWY	0.608	0.547	0.564	0.009	L-tryptophan biosynthesis
UDPNAGSYN-PWY	0.611	0.554	0.552	0.008	UDP-N-acetyl-D-glucosamine biosynthesis I
VALSYN-PWY	0.727	0.668	0.708	0.010	L-valine biosynthesis

Table 3. Major MetaCyc pathways (>0.5% at least one of the age-groups) by age groups in hand skin.

**Feature**	**Relative abundance (%)**			**SEM**	**Description**
	**10-29 years**	**30-49 years**	**50-79 years**		
1CMET2-PWY	0.510	0.503	0.519	0.010	N10-formyl-tetrahydrofolate biosynthesis
ANAEROFRUCAT-PWY	0.486	0.502	0.421	0.013	homolactic fermentation

ANAGLYCOLYSIS-PWY	0.543	0.580	0.580	0.034	glycolysis III (from glucose)
ARGSYN-PWY	0.481	0.488	0.519	0.011	L-arginine biosynthesis I (via L-ornithine)
ARGSYNBSUB-PWY	0.563	0.539	0.562	0.011	L-arginine biosynthesis II (acetyl cycle)
ARO-PWY	0.590	0.579	0.600	0.015	chorismate biosynthesis I
BRANCHED-CHAIN-AA-SYN-PWY	0.593	0.569	0.545	0.014	superpathway of branched amino acid biosynthesis
CALVIN-PWY	0.524	0.525	0.491	0.009	Calvin-Benson-Bassham cycle
COA-PWY	0.534	0.541	0.680	0.046	coenzyme A biosynthesis I
COMPLETE-ARO-PWY	0.613	0.577	0.587	0.008	superpathway of aromatic amino acid biosynthesis
DAPLYSINESYN-PWY	0.524	0.503	0.487	0.010	L-lysine biosynthesis I
DENOVOPURINE2-PWY	0.480	0.477	0.563	0.024	superpathway of purine nucleotides de novo biosynthesis II
DTDPRHAMSYN-PWY	0.378	0.405	0.531	0.042	dTDP-L-rhamnose biosynthesis I
FAO-PWY	0.562	0.535	0.494	0.022	fatty acid β-oxidation I
FASYN-ELONG-PWY	0.618	0.619	0.660	0.013	fatty acid elongation -- saturated
FASYN-INITIAL-PWY	0.520	0.544	0.590	0.019	superpathway of fatty acid biosynthesis initiation (E. coli)
FOLSYN-PWY	0.536	0.513	0.506	0.009	superpathway of tetrahydrofolate biosynthesis and salvage
GLUCONEO-PWY	0.511	0.508	0.467	0.009	gluconeogenesis I
GLYCOLYSIS	0.507	0.520	0.474	0.011	glycolysis I (from glucose 6-phosphate)
ILEUSYN-PWY	0.619	0.600	0.574	0.016	L-isoleucine biosynthesis I (from threonine)
NONOXIPENT-PWY	0.575	0.587	0.641	0.018	pentose phosphate pathway (non-oxidative branch)
OANTIGEN-PWY	0.421	0.435	0.506	0.017	O-antigen building blocks biosynthesis (E. coli)
PENTOSE-P-PWY	0.456	0.507	0.472	0.013	pentose phosphate pathway
PEPTIDOGLYCANSYN-PWY	0.543	0.565	0.610	0.025	peptidoglycan biosynthesis I (meso-diaminopimelate containing)
PHOSLIPSYN-PWY	0.710	0.727	0.761	0.018	superpathway of phospholipid biosynthesis I (bacteria)
POLYISOPRENSYN-PWY	0.521	0.547	0.605	0.025	polyisoprenoid biosynthesis (E. coli)
PWY-2942	0.590	0.604	0.606	0.017	L-lysine biosynthesis III
PWY-3001	0.555	0.536	0.516	0.011	superpathway of L-isoleucine biosynthesis I
PWY-3781	1.095	1.068	1.054	0.046	aerobic respiration I (cytochrome c)
PWY-5097	0.497	0.504	0.465	0.011	L-lysine biosynthesis VI

PWY-5101	0.657	0.622	0.608	0.016	L-isoleucine biosynthesis II
PWY-5103	0.569	0.542	0.520	0.014	L-isoleucine biosynthesis III
PWY-5484	0.502	0.515	0.451	0.013	glycolysis II (from fructose 6-phosphate)
PWY-5659	0.494	0.502	0.498	0.014	GDP-mannose biosynthesis
PWY-5667	0.783	0.799	0.864	0.020	CDP-diacylglycerol biosynthesis I
PWY-5686	0.608	0.620	0.657	0.022	UMP biosynthesis
PWY-5695	0.661	0.665	0.653	0.017	urate biosynthesis/inosine 5'-phosphate degradation
PWY-5971	0.495	0.509	0.593	0.019	palmitate biosynthesis II (bacteria and plants)
PWY-5973	0.637	0.704	0.762	0.024	cis-vaccenate biosynthesis
PWY-5989	0.580	0.566	0.595	0.017	stearate biosynthesis II (bacteria and plants)
PWY-6121	0.628	0.626	0.789	0.049	5-aminoimidazole ribonucleotide biosynthesis I
PWY-6122	0.608	0.610	0.776	0.050	5-aminoimidazole ribonucleotide biosynthesis II
PWY-6123	0.533	0.537	0.595	0.023	inosine-5'-phosphate biosynthesis I
PWY-6125	0.598	0.602	0.635	0.023	superpathway of guanosine nucleotides de novo biosynthesis II
PWY-6126	0.615	0.625	0.666	0.023	superpathway of adenosine nucleotides de novo biosynthesis II
PWY-6147	0.525	0.484	0.467	0.015	6-hydroxymethyl-dihydropterin diphosphate biosynthesis I
PWY-6163	0.566	0.559	0.618	0.019	chorismate biosynthesis from 3-dehydroquinate
PWY-6277	0.608	0.610	0.776	0.050	superpathway of 5-aminoimidazole ribonucleotide biosynthesis
PWY-6282	0.566	0.557	0.594	0.016	palmitoleate biosynthesis I (from (5Z)-dodec-5-enoate)
PWY-6385	0.534	0.563	0.599	0.023	peptidoglycan biosynthesis III (mycobacteria)
PWY-6386	0.539	0.554	0.589	0.021	UDP-N-acetylmuramoyl-pentapeptide biosynthesis II (lysine-containing)
PWY-6387	0.545	0.568	0.604	0.024	UDP-N-acetylmuramoyl-pentapeptide biosynthesis I (meso-diaminopimelate containing)
PWY-6612	0.512	0.489	0.475	0.010	superpathway of tetrahydrofolate biosynthesis
PWY-6628	0.505	0.484	0.485	0.011	superpathway of L-phenylalanine biosynthesis
PWY-6897	0.476	0.482	0.541	0.023	thiamin salvage II
PWY-6969	0.512	0.483	0.386	0.017	TCA cycle V (2-oxoglutarate:ferredoxin oxidoreductase)
PWY-7094	0.579	0.523	0.533	0.025	fatty acid salvage
PWY-7111	0.805	0.734	0.700	0.027	pyruvate fermentation to isobutanol (engineered)

PWY-7184	0.581	0.583	0.614	0.022	pyrimidine deoxyribonucleotides de novo biosynthesis I
PWY-7197	0.502	0.504	0.546	0.021	pyrimidine deoxyribonucleotide phosphorylation
PWY-7208	0.721	0.737	0.787	0.025	superpathway of pyrimidine nucleobases salvage
PWY-7211	0.522	0.504	0.515	0.009	superpathway of pyrimidine deoxyribonucleotides de novo biosynthesis
PWY-7219	0.622	0.635	0.782	0.048	adenosine ribonucleotides de novo biosynthesis
PWY-7220	0.610	0.621	0.634	0.023	adenosine deoxyribonucleotides de novo biosynthesis II
PWY-7221	0.608	0.619	0.663	0.024	guanosine ribonucleotides de novo biosynthesis
PWY-7222	0.610	0.621	0.634	0.023	guanosine deoxyribonucleotides de novo biosynthesis II
PWY-7228	0.623	0.626	0.664	0.024	superpathway of guanosine nucleotides de novo biosynthesis I
PWY-7229	0.644	0.657	0.705	0.024	superpathway of adenosine nucleotides de novo biosynthesis I
PWY-7234	0.521	0.527	0.583	0.024	inosine-5'-phosphate biosynthesis III
PWY-7254	0.553	0.546	0.499	0.019	TCA cycle VII (acetate-producers)
PWY-7400	0.478	0.485	0.514	0.011	L-arginine biosynthesis IV (archaebacteria)
PWY-7539	0.545	0.498	0.480	0.015	6-hydroxymethyl-dihydropterin diphosphate biosynthesis III (Chlamydia)
PWY-7663	0.708	0.746	0.829	0.025	gondoate biosynthesis (anaerobic)
PWY-7664	0.578	0.573	0.610	0.016	oleate biosynthesis IV (anaerobic)
PWY-841	0.602	0.605	0.661	0.024	superpathway of purine nucleotides de novo biosynthesis I
PWY0-1319	0.783	0.799	0.864	0.020	CDP-diacylglycerol biosynthesis II
PWY0-1586	0.609	0.696	0.700	0.029	peptidoglycan maturation (meso-diaminopimelate containing)
PWY0-162	0.610	0.618	0.653	0.022	superpathway of pyrimidine ribonucleotides de novo biosynthesis
PWY0-166	0.546	0.550	0.589	0.021	superpathway of pyrimidine deoxyribonucleotides de novo biosynthesis (E. coli)
PWY0-862	0.548	0.544	0.579	0.015	(5Z)-dodec-5-enoate biosynthesis
PWY4FS-7	0.670	0.684	0.711	0.018	phosphatidylglycerol biosynthesis I (plastidic)
PWY4FS-8	0.670	0.684	0.711	0.018	phosphatidylglycerol biosynthesis II (non-plastidic)
PWYG-321	0.618	0.574	0.655	0.020	mycolate biosynthesis
REDCITCYC	0.564	0.521	0.502	0.021	TCA cycle VIII (helicobacter)
SALVADEHYPOX-PWY	0.525	0.525	0.487	0.020	adenosine nucleotides degradation II
SER-GLYSYN-PWY	0.605	0.582	0.613	0.016	superpathway of L-serine and glycine biosynthesis I

TCA	0.644	0.613	0.572	0.020	TCA cycle I (prokaryotic)
THRESYN-PWY	0.531	0.523	0.581	0.015	superpathway of L-threonine biosynthesis
TRNA-CHARGING-PWY	0.521	0.519	0.507	0.008	tRNA charging
TRPSYN-PWY	0.512	0.489	0.458	0.014	L-tryptophan biosynthesis
UDPNAGSYN-PWY	0.553	0.553	0.602	0.021	UDP-N-acetyl-D-glucosamine biosynthesis I
VALSYN-PWY	0.619	0.600	0.574	0.016	L-valine biosynthesis
